# Anæsthesia—Nitrous Oxide

**Published:** 1881-11

**Authors:** Geo. Watt


### ANÆSTHESIA—NITROUS OXIDE.

BY GEO. WATT.

In the consideration of the various agents used in the production of anæsthesia, many reasons might be given for placing this one first in the list. The first decided, practical success in anæsthesia was obtained by the use of this agent. It is used oftener than all the others.

In view of his many and interesting experiments with nitrous oxide, we are surprised that Sir Humphrey Davy did not develop the subject of anæsthesia, in all its practical utility, just as we are surprised that James Watt did not invent the railroad as soon as he had completed the steam engine. Davy's failure to complete the discovery is doubtless due to the very small quantity of gas used in his experiments, and to the fact that the breath was re-inhaled with the gas. Partial suffocation of the patient was a necessary accompaniment of such experiments. A remark of Regnault in this direction, throws some light on the inefficiency of early experiments with this agent. He is of much later date than Davy, and he cautions experimenters against the suffocating methods of early chemists. He says a sack to contain the gas for inhaling should be large, and not smaller than the bladder of an ox. And into this he expected the breath to be exhaled and re-inhaled, again and again, and yet such botch-work was called experiments with nitrous oxide; and, in the revival of nitrous oxide, after the long disuse of it, following the death of Wells, Mr. Colton, and the dental prefession as well, showed but little, if any more common sense. Rubber bags had become common, and hence bladders were not re-introduced. But think of small gas-bags, of three or four gallons each, laid on the laps of refined, delicate ladies, tubes inserted into their mouths, their lips compressed against them, their nostrils closed by screw-clamps and the poor victims left to die at once of apnœa, or re-inhale, over and over again, the filthiest and deadliest of all their own excrements, to say nothing of those already in the bags, left by the rotten, syphilitic pimps and prostitutes who had preceded them as victims. Rabshakeh's proposition to Hezekiah's Hebrew soldiers was a polite and cleanly suggestion in comparison. Is the picture overdrawn? Let words bear witness: At the meeting of the Mississippi Valley Association for 1864, one member, a leading man in the profession then and now, said, "a four-gallon gas bag would be usually found sufficient for three inhalations." He was understood that such a bag, full of gas, would prove sufficient for three operations on as many patients. And further, he said, "he had administered the gas to three different patients from the same bag, without renewing." The writer of this objected to such course at the time, but the statement did not seem to make a sensation. Another member remarked, "by using the gas-bag too often, without replenishing, he had seen suffocation result before anæsthesia." And, not far from the same date, Mr. Colton expressed the opinion that re-inhaling from a gas-bag was preferable to the use of a valved inhaler, as the carbonic acid of the breath appeared to be necessary to complete anæsthesia. All the older members of our profession will remember that this filthy method was in common use. The writer of this cannot yet think of the inside of a bag so used, without suffering from nausea.

COMPOSITION OF NITROUS OXIDE.

This gas is the protoxide of nitrogen, that is, it is composed of fourteen parts, by weight, of nitrogen, united with eight parts of oxygen. Its symbol is, therefore, NO, by the old notation. That it is a combination of its elements, and not a mere mixture, is evident from the fact that it is possessed of taste and smell, while its components are both tasteless and inodorous.

THE OXIDES OF NITROGEN.

Nitrogen is liable to various degrees of oxidation, and each degree results in a compound radically differing from all the others. There is *nitrous oxide*, already described; *nitric oxide*, composed of sixteen parts of oxygen, combined with fourteen of nitrogen; *hyponitrous acid*, in which twenty-four parts of oxygen unite with fourteen of nitrogen; *nitrous acid*, thirty-two of oxygen with the fourteen of nitrogen; *nitric acid*, composed of forty parts of oxygen combined with fourteen of nitrogen. The latter is supposed to be the highest degree of oxidation of nitrogen, or, it may be said, the combustion of nitrogen is complete. These compounds differ in their characteristics to a remarkable degree. All but the first are very energetic chemical reagents. Much of this energy in the second, third and fourth is due to their inordinate demand for additional quantities of oxygen. The chemical energy of the fifth is due in part to its great affinity for basic oxides, water included, and, in part to the readiness with which it parts with portions of its oxygen, to form these bases, that the residue as above described, may combine with them. The salts thus formed are called *nitrates*. The special activities of these compounds are here explained because both physicians and denists have argued that the first-named, (nitrous oxide) must be dangerous because allied to the other oxides of nitrogen, which possess dangerous properties. But no argument could be more fallacious.

Nitrous oxide, unlike its comrades, is not demanding more oxygen, but, on the contrary, it is ever ready to give up, on the slightest pretexts, that which it already has. The affinity holding its component elements together, is so feeble that a spark is re-kindled into flame as readily in nitrous oxide as in free oxygen. And after the re-kindling the combustion is all the more energetic in the former, because the oxygen is nascent, that is, it is oxygen as ozone. That a spark may be re-kindled into flame in nitrous oxide, the gas must be decomposed, for, as nitrous oxide, it is a non-supporter of combustion. And the readiness of decomposition is so marked that no one can see that a spark is not as promptly re-kindled by it as by free oxygen. And it is this readiness of decomposition that enables it to support respiration for a time, as it readily does. The continued breathing of it irritates the air passages, because the oxygen is active, being nascent instead of passive, as is most of that in the atmosphere.

It is very unfortunate that when a man greatly excels his fellows in some branches of knowledge, he often forgets that he does not excel in all; and a consequence of this misfortune is, that he often speaks with as much assurance on subjects of which he knows nothing, as he does on those which he has fully mastered. The masses of the profession recognize the superiority of such a man, on some subjects about which they know a little, and, of course, with his positive assertions, they take him on trust, on subjects about which both he and they know nothing. This trait of the human mind enables a man of positive character and strong self-assertion to do great mischief. The writer of this has had to listen to such, who he knew had spent fewer minutes than he had months in the study of the subject, stating to others who had given the matter as little attention as themselves that nitrous oxide is not a better supporter of respiration than carbonic acid; that a human being would live as long under water as in an atmosphere of nitrous oxide, etc., when he could readily recall the time he had breathed it for an hour, at noon, and had done a full half-day's work in the afternoon, without discomfort then or afterward. At the same time he could recall the day that an English terrier had breathed the gas from 11 A. M. to 12 M., without any admixture of air, and remained so true to nature and function, that he killed over seventy rats between 1 P. M. and 2 P. M. of the same day.

But while such facts pertain to nitrous oxide, a single breath of nitric oxide, if it were possible to take it, would prove instantly fatal; and the difference lies in the facts that one gives out oxygen almost as readily as if free, yet quite too active for normal respiration, while the other takes oxygen direct from the red corpuscles, or any other tissue that contains it, and is, thereby converted into a caustic acid, capable of destroying any texture of the body, and this acid is presented in all the ferocious energy of its nascent state. The presence of minute traces of nitric oxide in the nitrous oxide constitutes the chief danger in the use of gas in dental surgery. On the other hand, there are silly advocates of nitrous oxide who maintain that, as it is composed of the same constituents as atmospheric air, and is richer in oxygen, it is, therefore, a better supporter of respiration than the atmosphere. But our Father in Heaven makes no mistakes, and he has not given us an atmosphere of the oxide. And it is a mere assumption, and not true, that it is composed of the same ingredients that constitute atmospheric air, for carbonic acid is an essential constituent of our atmosphere.

The fact that nitrous oxide is a medicine and not a plaything should never be forgotten, and, consequently, it should be used only by those who are well versed in the knowledge of the human constitution and in the science of chemistry. The habit of taking or administering nitrous oxide to see what disposition will be manifested under its influence, for the gratification of the curious, is not more legitimate nor sensible than it would be, under similar circumstances, to give doses of ipecacuanha, to see which of the victims would be the first to vomit. The most serious drawback in the way of the proper introduction of nitrous oxide into surgery, is the fact that, for more than half a century it has been used as a plaything, or means of amusement, by the ignorant and unscrupulous.

PREPARATION OF

Nitrous oxide is usually obtained by decomposing nitrate of ammonia by the aid of heat. In solution this salt is colorless, when solid it is white. It is composed of one equivalent of nitric acid and one of ammonia. Ammonia is one of the alkalies and is composed of fourteen parts of nitrogen combined with three of hydrogen. Its symbol (old style) is $NH_3$, while that of the acid is $NO_5$. This takes no account of the water of crystallization, as its consideration would not aid us in understanding the reactions which occur. Each of the three elements composing this salt is a gas when free. Their natural elasticity is overcome while they are held in combination to form the salt. Heat, therefore, decomposes the salt, by increasing their elasticity. When they are thus separated, other affinities assert themselves, and that between oxygen and hydrogen, being the strongest known in chemistry, is the key to the decomposition. When the two leaders have taken each other, it is easy to see what must become of the other elements. When the decomposition is effected as desired, the sole results are nitrous oxide and water; but it is doubtful if this is absolutely obtainable in practice, at least by present methods.

The salt is placed in a glass retort, or, what is better, a Florence flask, with a perforated rubber stopper, with glass tube attached. The heat is applied to the flask by means of a smokeless flame, a properly adjusted gas stove being preferable when practicable. The gas should be passed through three or four wash bottles or jars containing water. It is claimed by some that the addition of sulphate of iron to the first wash-bottle, and caustic potash or soda to one of the others, gives better results in the way of pure gas. The water should be renewed in the first bottle, each time, before using again. This bottle must not be full, as the water of crystallization, and that formed by chemical combination, during the process, are condensed in it. It is not necessary to give here a minute description of the details in making the gas, as those who expect to make their own should receive practical instruction from experienced chemists, before attempting the process in practice. Many, and perhaps a majority of those using the gas, buy it ready for use, condensed to a liquid form in strong cylinders. This puts them on a par, as to nitrous oxide, with surgeons in the use of ether and chloroform. They sink their own chemical responsibility in that of the manufacturer. And this is well in the present state of chemical knowledge in our profession. Thus far, however, we have preferred to prepare our own, and shall probably continue so to do.

It has been stated already, that when nitrate of ammonia is decomposed as desired, the sole results are water and nitrous oxide. This decomposition may be illustrated by an equation, thus:

$$NH_3,NO_5=3HO+2NO.$$

This equation represents one equivalent of nitrous oxide decomposed into three equivalents of water, and two of nitrous oxide. For the benefit of the non-chemical reader, it may be well to explain the equation. N, H and O respectively represent nitrogen, hydrogen, and oxygen. The letters are called symbols. The small figure to the right affects only the symbol next to it. The large figure, on the left, affects all the symbols between it and the next break in the line. When no figure stands at the side of a symbol, figure 1 is understood. Now look at the equation: $NH_3$ shows that nitrogen and hydrogen are present; standing together shows they are combined; no figure beside N shows that one equivalent is represented. The small figure 3 beside H shows that three equivalents of hydrogen are in the combination; but such a combination constitutes ammonia. In like manner the one equivalent of nitrogen and five of oxygen constitute nitric acid; and these two compound symbols, being separated by only a comma, give expression to the idea that they have combined. But when nitric *acid* combines with the *alkali*, ammonia, we have nitrate of ammonia. The right side of the equation is simpler: Figure 3 affects both H and O. But three equivalents of hydrogen combined with three of oxygen, give three of water. Nitrous oxide is composed of one equivalent each of nitrogen and oxygen, and the large figure 2 shows that we have two equivalents of this, the desired gas. Now, if the reader, who is not versed in chemical notation, will carefully study this explanation of this equation, he will be able to read and understand almost any chemical equation.

If the decomposition of nitrate of ammonia represented by the equation above were obtainable at will, with absolute mathematical accuracy, then would nitrous oxide be recognized as a blessing beyond estimation, both as an anæsthetic and as a therapeutic agent. No darkening of the complexion could occur during its administration, unless the operator should deliberately suffocate his patient, or victim, rather. But a doubt in this direction has been already expressed. With the decomposing heat too low, a little, and with it too high, an abundance of a deadly gas passes over with the nitrous oxide. In any decomposition which may occur, nitrous oxide is chiefly given off; but a very limited quantity of the poisonous gas referred to is capable of producing disastrous results. The deadly gas referred to is nitric oxide, which is just twice as rich in oxygen as is the nitrous. Its symbol is $NO_2$, and, when it is given off the order of decomposition is probably represented by the following equation:

$$NH_3 NO_5 = 2NO_2 + HO + H_2.$$

Should the decomposition of the salt occur, as represented by this equation, it must be borne in mind that a much more abundant decomposition in accordance with the preceding equation takes place at the same time. An experienced eye can often detect the latter decomposition by the peculiar appearance of the surface of the melted nitrate of ammonia in the flask or retort. This appearance is due to an infinitesimal series of minute explosions caused by the coming together of free hydrogen and nitrous oxide. How and why the nitric oxide is so deadly in its effects on the constitution must be left to another paper. Suffice it to say that with security that it shall not be present, the use of nitrous oxide in dental surgery is almost robbed of its dangers,—indeed entirely so, if the operator is intelligent.

It is not so easy to get rid of nitric oxide, as many imagine, when it has become commingled with nitrous oxide. The poisonous gas is invisible, colorless, tasteless, about as heavy as atmospheric air. Some have claimed that, as it is about a third lighter than nitrous oxide, it will rise above the latter and may be drawn off; but such have only shown their ignorance of the laws of gaseous diffusion. Many have claimed that it can be washed out by passing through, or preserving over water, the mixture of gases. They would scarcely try to wash sand out of pulverized sugar in the same way, yet the two processes are similar; that is, the nitrous is far more soluble in water than nitric oxide. Water dissolves about its own volume of the former, and only eleven per cent. of the latter. It would be easy, therefore, to wash nitrous out of nitric oxide, while to reverse the process would be found utterly impracticable. Atmospheric air changes nitric oxide to nitrous acid, which can be easily washed out with water; but as we do not know how much nitric oxide is present where we wished and intended to have none, so we do not know how much air to admit in any given case. If we admit too much our gas is diluted; if too little, the poisonous gas is still present to a greater or less extent. The proper way, if practicable, is to not make nitrous oxide.

*Ohio State Journal Dental Science.*

